# Spiny neurons of amygdala, striatum, and cortex use dendritic plateau potentials to detect network UP states

**DOI:** 10.3389/fncel.2014.00292

**Published:** 2014-09-17

**Authors:** Katerina D. Oikonomou, Mandakini B. Singh, Enas V. Sterjanaj, Srdjan D. Antic

**Affiliations:** Department of Neuroscience, University of Connecticut Health CenterFarmington, CT, USA

**Keywords:** NMDA spike, dendritic plateau potentials, dendritic spike, voltage-sensitive dye imaging, UP states, amygdala, striatum

## Abstract

Spiny neurons of amygdala, striatum, and cerebral cortex share four interesting features: (1) they are the most abundant cell type within their respective brain area, (2) covered by thousands of thorny protrusions (dendritic spines), (3) possess high levels of dendritic NMDA conductances, and (4) experience sustained somatic depolarizations *in vivo* and *in vitro* (UP states). In all spiny neurons of the forebrain, adequate glutamatergic inputs generate dendritic plateau potentials (“dendritic UP states”) characterized by (i) fast rise, (ii) plateau phase lasting several hundred milliseconds, and (iii) abrupt decline at the end of the plateau phase. The dendritic plateau potential propagates toward the cell body decrementally to induce a long-lasting (longer than 100 ms, most often 200–800 ms) steady depolarization (∼20 mV amplitude), which resembles a neuronal UP state. Based on voltage-sensitive dye imaging, the plateau depolarization in the soma is precisely time-locked to the regenerative plateau potential taking place in the dendrite. The somatic plateau rises after the onset of the dendritic voltage transient and collapses with the breakdown of the dendritic plateau depolarization. We hypothesize that neuronal UP states *in vivo* reflect the occurrence of dendritic plateau potentials (dendritic UP states). We propose that the somatic voltage waveform during a neuronal UP state is determined by dendritic plateau potentials. A mammalian spiny neuron uses dendritic plateau potentials to detect and transform coherent network activity into a ubiquitous neuronal UP state. The biophysical properties of dendritic plateau potentials allow neurons to quickly attune to the ongoing network activity, as well as secure the stable amplitudes of successive UP states.

## INTRODUCTION

### NEURONAL UP STATES

#### The binding theory

**FIGURE 1:**
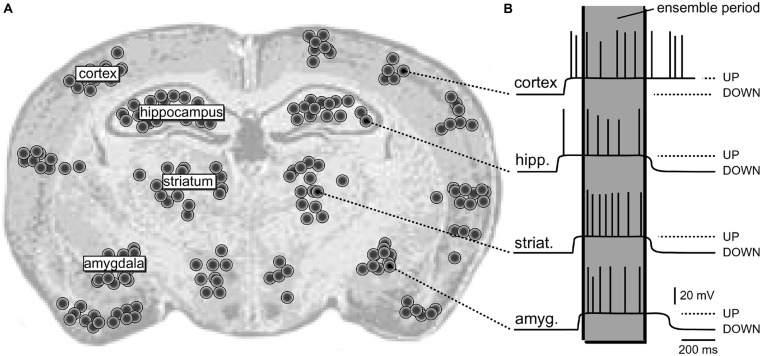
**Schematic depiction of a neural ensemble (hypothetical). (A)** Coronal section of a rat brain. One hypothetical neural ensemble is comprised of neurons synchronously experiencing suprathreshold depolarizations. These neurons are distributed unevenly across several brain regions, including cortical layers 2–6, neostriatum, hippocampus, and amygdala. Each black dot denotes 50 neurons. **(B)** Intracellular recordings show a plateau depolarization (UP state) crowned by action potentials (APs). Note that neurons do not fire APs in the DOWN state. Also, the onsets and offsets of UP states are not perfectly synchronized. However, in one brief period of time (marked by the gray column – “ensemble period”) all relevant neurons are in the UP state; they all joined the dynamic neural ensemble.

The dynamic structure composed of synchronously activated neurons engaged in the same task is termed “neural ensemble” (Hebb, 1949; Eichenbaum, 1993; Engel and Singer, 2001). Individual members of a “neural ensemble” are widely distributed across different areas of the brain (**Figure 1A**), each specialized in signaling a different attribute of the object or different element within the scene (Perrett et al., 1982; Mountcastle, 1997; Singer, 1999; Yu and Ferster, 2010). Proper representation of a physical or mental “object” during sensory perception requires the “binding” together of many attributes into a single experience. “Binding” is simply a synchronization of electrical activity of large populations of neurons on a definite temporal scale (**Figure 1B**).

#### Dynamic ensembles

The dynamic feature of information processing in the brain is reflected in the fact that at one instant of time any given neuron is a member of one ensemble, while in the next instant of time the same neuron participates meaningfully in the function of another neuronal ensemble (Desimone et al., 1984; Eichenbaum, 1993; Wilson and McNaughton, 1993; Engel and Singer, 2001). This “time-sharing” feature of the ensemble-organization principle assures virtually an infinite number of neuronal ensembles in the mammalian brain that can be assigned to an infinite number of specific objects, including perceptual and mental objects.

#### Neural synchronization

Synchronized spiking activity has been found in different species and different cortical areas (Bair, 1999; Salinas and Sejnowski, 2001; Buzsaki and Silva, 2012). For the same level of firing, a synchronous input is more effective on postsynaptic neurons than asynchronous input (Schneidman et al., 1998; London et al., 2002). Large-scale models predict that synchrony occurs due to the reciprocal connectivity and loops between clumps of neurons (Tononi et al., 1992; Durstewitz et al., 2000; Compte et al., 2003). It is tempting to state that oscillatory activity and phase alignment between distant groups of neurons is the preferred mechanism of the “*Binding theory*” (Engel and Singer, 2001; Tononi and Koch, 2008; Ainsworth et al., 2012). Oscillatory activity, even when subthreshold, could facilitate synchronous interactions by biasing neurons to discharge within the same time frame (Engel et al., 2001; Yu and Ferster, 2010; Petersson and Fransen, 2012). The main effect of the oscillatory modulation of neuronal membrane potential is that it constrains the time interval during which nerve cells are susceptible to excitatory input and can reliably emit bursts of action potentials (**Figure 1B**). In this paper we will argue that glutamate-mediated dendritic plateau potentials provide such time intervals.

#### Time window 200–500 ms

The majority of brain processes related to the feeling of awareness require that neural activity lasts for 200–500 ms (Libet et al., 1979). This window of time is perhaps a minimum amount of time needed to guarantee interactions among multiple brain regions. The 200–500 ms of sustained firing triggers the awareness of a stimulus either directly by producing significant glutamatergic output in target brain areas, or indirectly by allowing the feedforward stream from thalamus to interact appropriately with feedback stream from higher cortical areas (Cauller, 1995; Lamme and Roelfsema, 2000; Engel et al., 2001; Ro et al., 2003; Larkum, 2012). The 200–500 ms time window of sustained neuronal depolarization may be the consequence of reverberant activity closing the loop between past and present features of a moving object, or by closing the loop between long-term memory traces and the current sensory percept (reviewed in Tononi and Koch, 2008). Interestingly, the duration of synaptically evoked dendritic plateau potentials is also in the range of 200–500 ms (Milojkovic et al., 2004; Oikonomou et al., 2012).

#### Spiny neurons

**FIGURE 2:**
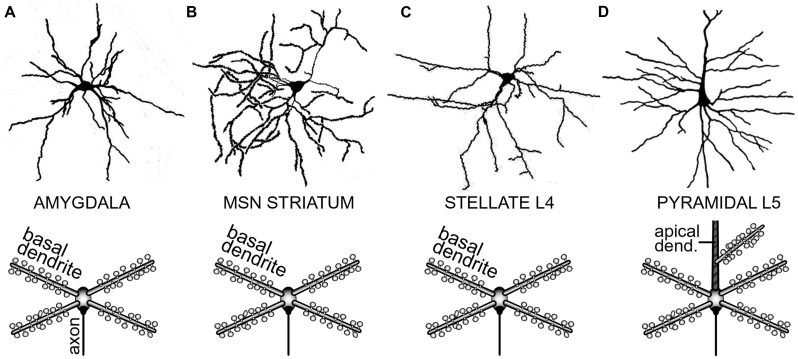
**Determinants of dendritic morphology in spiny neurons of the forebrain. (A)** Spiny neuron in the amygdaloid complex. In this and all the remaining panels, upper image is a camera lucida drawing, while the lower image is a conceptual representation of the dendritic tree. Upper image adopted from (Shaikh and Shaikh, 1982). **(B)** Medium spiny neuron (MSN) of the neostriatum. Upper image adopted from (Klapstein et al., 2001). **(C)** Spiny stellate cell in cortical layer 4. Upper image adopted from (Andjelic et al., 2009). **(D)** Cortical layer 5 pyramidal neuron. Upper image adopted from Gray’s Anatomy, p. 722. Major morphological distinction of L5 pyramidal neurons is the presence of an apical dendrite (striped area). Basilar dendrites are the common feature of all spiny neurons.

**FIGURE 3:**
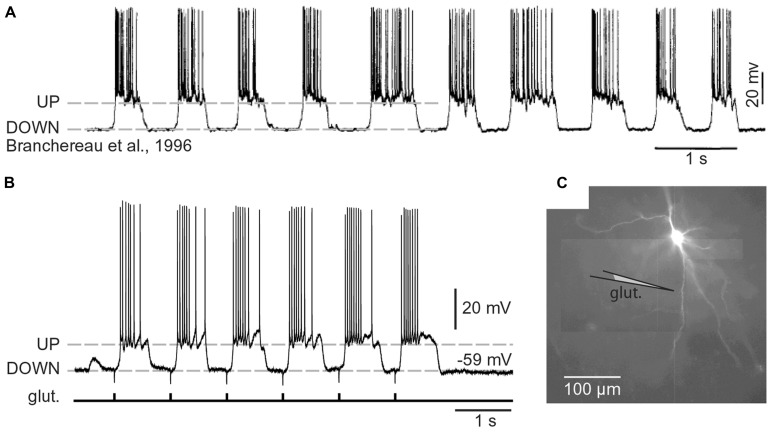
**Cortical UP and DOWN states. (A)** *In vivo* intracellular recording from a pyramidal neuron in the rat medial prefrontal cortex. Adapted from Branchereau et al. (1996). **(B)** *In vitro* whole-cell recording from a pyramidal neuron in the rat medial prefrontal cortex (brain slice). Glutamate pulses (duration 5 ms) were delivered every second on a basal dendritic branch, at a distance of 105 μm from the cell body. Dashed line marks the resting potential (-59 mV). Note that the slow component of somatic depolarization alternates between depolarized (UP) and hyperpolarized (DOWN) level. **(C)** Composite microphotograph of a rhodamine-filled neuron. Schematic drawing marks the position of the glutamate stimulation pipette on a basal dendrite. Adapted from Antic et al. (2007).

The distribution of neurons involved in one functional neural ensemble is not restricted to the cerebral neocortex, but it is likely to include subcortical gray matter (**Figure 1A**; Brecht et al., 1998; Ziaei et al., 2013). In brain regions strongly implicated in cognition and memory formation (neocortex, thalamus, neostriatum, ventral striatum, amygdala, and hippocampus), the principle and/or most numerous neurons are those that have protoplasmatic protrusions termed “dendritic spines” (Nimchinsky et al., 2002). During non-REM slow-wave sleep, spiny neurons experience 1 Hz fluctuations in membrane potential (UP and DOWN states), as documented by *in vivo* intracellular recordings (Volgushev et al., 2006). The spontaneous plateau depolarizations (UP states) are ~20 mV in amplitude and several 100 ms in duration. The UP states may or may not be accompanied by action potential firing (O’Donnell and Grace, 1995; Branchereau et al., 1996; Contreras et al., 1996; Wilson, 2008). *In vivo* intracellular recordings have documented UP and DOWN transitions in cortical L5 pyramidal neurons, cortical L4 stellate cells, striatal medium spiny neurons and spiny neurons of the amygdala (Wilson and Kawaguchi, 1996; Steriade et al., 2001; Brecht and Sakmann, 2002; Volgushev et al., 2006; Padival et al., 2013). These four neuron types differ in many respects including their fine morphology, developmental origin, wiring, and immunohistochemical markers. However, viewed from a purely biophysical aspect, all four aforementioned neuron subtypes exhibit identical plan of organization, except for the addition of one apical dendrite to the pyramidal neurons (**Figure 2**). It can be said that the basilar dendritic tree is a common feature of all spiny neurons (**Figure 2**). Benucci et al. (2004) manipulated the gross morphological structure of cortical pyramidal and neostriatal MSNs cells in realistic multicompartmental models. Benucci et al. (2004) kept the morphology of the basal dendritic tree unchanged, but reduced the apical part of a pyramidal neuron to a single equivalent compartment. Despite of this drastic morphological modification, the qualitative aspects of the bimodal intracellular dynamics (UP and DOWN states) were preserved (Benucci et al., 2004). Benucci et al. (2004) concluded that an intact basal dendritic tree is the minimal condition necessary for the emergence of UP and DOWN states. In support of this conclusion, *in vitro* electrophysiological experiments performed in cortical pyramidal cells showed that brief (5 ms) glutamate pulses delivered on a single basal branch produce long-lasting somatic plateau depolarizations, which resemble neuronal UP states *in vivo* (**Figure 3**; Branchereau et al., 1996; Wilson, 2008).

Spiny CNS neurons (pyramidal and MSN neurons) rarely fire action potentials from a DOWN state (**Figure 3A**). A successful synchronization of the firing activity among neurons would require that members of a neuronal ensemble enter UP state at the same moment of time (**Figure 1B**). To become eligible for inclusion into a functional neuronal ensemble, a spiny neuron must quickly, and reliably switch from a DOWN to an UP state, remain in the depolarized UP state as long as necessary, and quickly abort the UP state when a percept is formed or expired. Although the focus of this manuscript is on glutamatergic transmission, one should not ignore that the great majority of GABAergic inputs impinge directly on the dendrites of cortical and sub-cortical principal neurons and may profoundly influence the dendritic processing of glutamatergic inputs (Gidon and Segev, 2012), which in turn may impact the onset and offset of neuronal UP states (Shu et al., 2003; Windels et al., 2010).

#### Cellular bases of UP states

Several competing theories aim to explain the cellular bases of UP and DOWN states. The first hypothesis stated that spontaneous transmitter release occurring during a DOWN state occasionally depolarizes certain cells to the firing threshold, thus initiating an active state in the network (Timofeev et al., 2000; Bazhenov et al., 2002). The “spontaneous release” hypothesis predicts that cells receiving largest excitatory convergence will have the highest probability of being activated before other cells in the network (Chauvette et al., 2010). Note that spiny neurons are cells with the largest excitatory convergence in any given network. The second hypothesis suggests that UP states are mediated by intrinsic oscillations of layer 5 pyramidal neurons. The “intrinsic oscillation” hypothesis predicts that once initiated by layer 5 neurons, activity then propagates to other cortical layers (Sanchez-Vives and McCormick, 2000). The third hypothesis attributes transitions from silent to active states to the selective synchronization of neuronal ensembles involving a small number of “pacemaker” cells grouped in a cluster. The “pacemaker cluster” hypothesis predicts a very stereotyped spatiotemporal dynamics of UP state triggering (Cossart et al., 2003). In this paper we propose that dendritic plateau potentials occur in principal neurons during network UP states and are responsible for voltage waveforms regularly observed in the cell body using intracellular *in vivo* recordings (Timofeev et al., 2000; Chauvette et al., 2010). The relation between dendritic plateau potentials and UP states can be both causal and correlative. In the causal relation, a dendritic plateau potential triggers an UP state in one neuron, which in turn recruits other neurons to form a local network UP state. In the correlative relation, dendritic plateau potentials are caused by network UP states, given that the network UP states provide sufficient glutamatergic drives congregated onto one dendritic segment. In either case, causative or correlative, dendritic plateau potentials produce characteristic sustained depolarizations of the neuronal cell body during the UP states (Milojkovic et al., 2007; Augustinaite et al., 2014).

### DENDRITIC PLATEAU POTENTIALS

#### Glutamate-mediated dendritic spike

The voltage waveforms of glutamate-mediated dendritic spikes (Schiller et al., 2000) were characterized using voltage-sensitive dye imaging (Milojkovic et al., 2004, 2005a,b) and dendritic patch (Nevian et al., 2007; Larkum et al., 2009). Dendritic voltage-sensitive dye imaging revealed that the somatic plateau rises a few milliseconds after the onset of the dendritic voltage transient and collapses with the breakdown of the dendritic plateau depolarization (Milojkovic et al., 2005a). The slow component of the somatic depolarization accurately mirrors the glutamate-evoked dendritic plateau potential (dendritic UP state). This observation is most apparent in experiments in which a gradually increasing intensity of glutamatergic input was delivered onto a basilar dendritic branch. At subthreshold glutamate input intensities the dendritic and somatic depolarizations are both subthreshold. As soon as the dendritic membrane develops a regenerative dendritic plateau potential (Milojkovic et al., 2004, 2005a), the somatic compartment of this neuron reports a neuronal UP state (Oikonomou et al., 2012, their Figure 3). In summary, the relation between dendritic plateau potential and somatic UP state is uniquely reliable and faithful (Milojkovic et al., 2004, 2005a,b, 2007).

#### Dendritic NMDA spikes versus dendritic plateau potentials – differences

Glutamate-mediated dendritic plateau potentials can be distinguished from classic dendritic NMDA spikes based on:

***Duration.*** The half-widths (durations) of NMDA spikes are in the range of 15–50 ms. The half-widths of plateau potentials are greater than 100 ms, often in the range 200–500 ms. Notably, the dendrite will stay in the plateau phase as long as glutamate is present in the extracellular space (Milojkovic et al., 2005a; Oikonomou et al., 2012). Glutamate remains bound to the NMDA receptors because there is a surplus of glutamate in the extracellular space (**Figure 9**, glutamate pond).

***Amplitude.*** The somatic amplitude of a dendritic NMDA spike is not sufficient to trigger AP firing in healthy neurons at rest (Schiller et al., 2000; Polsky et al., 2004, 2009; Chalifoux and Carter, 2011; Oikonomou et al., 2012). Whereas the somatic amplitude of the dendritic plateau potential is a successful trigger of neuronal AP firing in ~90% of trials (Milojkovic et al., 2004, 2005a,b; Major et al., 2008).

***Ca^2+^ Map.*** During an NMDA spike the dendritic calcium influx is highly restricted to the excitatory input site (Schiller et al., 2000). During a glutamate-mediated dendritic-plateau potential the calcium flux engulfs the entire length of the respective dendritic branch (Milojkovic et al., 2007). While the influx of calcium at glutamate input site is solely due to the opening of NMDA receptor channels (Schiller et al., 2000), the influx of calcium in dendritic segments away from the glutamate input site is due to the propagation of plateau potential along dendritic cable, resulting in the activation (opening) of voltage gated calcium channels (**Figure 8**; Milojkovic et al., 2007).

***Synaptic Requirement.*** Synaptic stimulation (synaptic shock) is a standard procedure in cellular neuroscience used to evoke release of neurotransmitters from axon terminals by applying a brief (0.1 ms) current pulse via a stimulation electrode positioned near the afferent axons (**Figures 5A1,B1**). The major practical distinction between NMDA spikes and dendritic UP states (plateau potentials) lies primarily in the fact that NMDA spikes can readily be triggered by two synaptic shocks (Polsky et al., 2004; Chalifoux and Carter, 2011) and sometimes even one synaptic shock is sufficient (Milojkovic et al., 2004, their Figure 7). Dendritic plateau potentials, on the other hand, require repetitive synaptic stimulation; more than two synaptic shocks (Milojkovic et al., 2004; Oikonomou et al., 2012).

In summary, these four parameters (duration, amplitude, spatial distribution of calcium signal, and dependence on more than two consecutive excitatory inputs) can be used to distinguish between dendritic NMDA spike and glutamate-mediated plateau potential (Oikonomou et al., 2012, their Figure 2).

#### Dendritic NMDA spikes and dendritic plateau potentials – similarities

Dendritic NMDA spikes and Dendritic Plateau Potentials share several properties, such as:

***Ionic Composition.*** Both NMDA spikes and dendritic plateau potentials strongly depend on dendritic NMDA current (Schiller et al., 2000; Milojkovic et al., 2005a; Major et al., 2013; Augustinaite et al., 2014). Dendritic plateau potentials initially start as NMDA spikes, but their dynamics/waveform change significantly upon stronger (or repetitive) synaptic stimulation (Milojkovic et al., 2004, 2005a; Major et al., 2008).

***Somatic Depolarization.*** Both types of dendritic potentials produce somatic depolarizations significantly greater in amplitude than the conventional EPSPs. However, upon conversion from NMDA spike to dendritic plateau potential, the somatic voltage waveform is no longer like a large, pointy EPSP (Polsky et al., 2004, 2009; Oikonomou et al., 2012; Brandalise and Gerber, 2014); it becomes a more sustained depolarization event, reminiscent of a cortical UP state (Milojkovic et al., 2004, 2005a).

***Calcium Influx.*** Both types of dendritic potentials produce strong calcium accumulation at the glutamate input site. However, upon conversion from NMDA spike to dendritic plateau potential, the dendritic calcium signal switches from a highly localized calcium transient characteristic of NMDA spikes (Schiller et al., 2000; Holthoff et al., 2004; Chalifoux and Carter, 2011; Katona et al., 2011) to a robust calcium flux that engulfs the entire dendritic branch (Milojkovic et al., 2007; Major et al., 2008).

***Synaptic Requirement.*** Generation of NMDA spikes and glutamate-mediated dendritic plateau potentials can be achieved by any type of stimulation which brings substantial quantities of glutamate to synaptic and extrasynaptic NMDA receptors at the same time. Both NMDA spikes and plateau potentials can be triggered by repetitive synaptic stimulation (Milojkovic et al., 2004; Polsky et al., 2004; Oikonomou et al., 2012) or focal application of exogenous glutamate (Schiller et al., 2000; Milojkovic et al., 2005a; Losonczy et al., 2008; Chalifoux and Carter, 2011).

**FIGURE 4:**
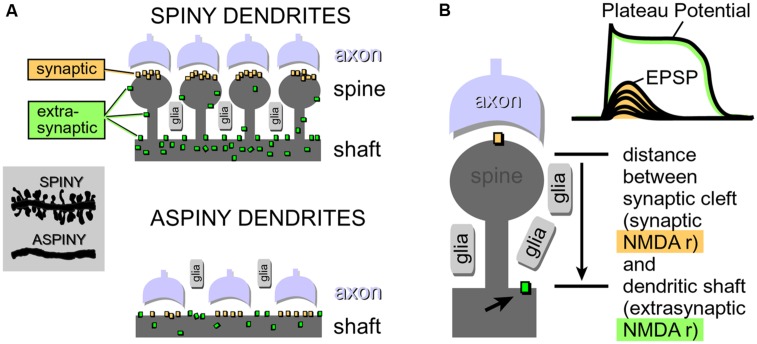
**Physical aspects of glutamatergic transmission in spiny and aspiny neurons. (A)** Dendritic spines increase the receptive area for impinging axons, resulting in a greater density of synaptic contacts in spiny neurons compared to aspiny neurons. For the same reason (increased receptive area), the total number of NMDA receptors per unit length is also greater in spiny neurons. Synaptic NMDA receptors are activated during all modes of synaptic transmission. Extrasynaptic NMDA receptors, on the other hand, are mostly activated by glutamate spillover during barrages of (repetitive) synaptic inputs. **(B)** If glutamate breaches the distance between synaptic cleft (synaptic) and the surface of the dendritic shaft (extrasynaptic), then subthreshold potential (EPSP) converts into a suprathreshold potential (Plateau Potential). Arrow points to an extrasynaptic NMDA receptor.

***Neuron types supporting NMDA spikes and glutamate-mediated dendritic plateau potentials.*** Both NMDA spikes and plateau potentials can be triggered in thin spiny dendrites of pyramidal neurons (basal, tuft, oblique), and not so successfully in aspiny segments of the thick apical dendrite (Schiller and Schiller, 2001; Larkum et al., 2009). This is probably due to the fact that the presence of dendritic spines (**Figure 4**) effectively increases two important factors: (a) the number of presynaptic glutamatergic terminals impinging on the dendritic segment; and (b) the number of postsynaptic glutamate receptors exposed to synaptic and extrasynaptic glutamate (Rusakov and Kullmann, 1998; Chalifoux and Carter, 2011; Oikonomou et al., 2012). Because NMDA spikes strongly depend on the density of NMDA receptor channels on spine heads, spine necks and dendritic shafts between dendritic spines (**Figure 4**), the ability of a dendrite to support an NMDA spike is a tell-tale sign of the ability of that dendrite to also generate dendritic UP states (plateau potentials). We searched for NMDA spikes in four neuron subtypes including spiny neurons of the amygdala (*n* = 24 neurons), striatal medium spiny neurons (*n* = 12 neurons), stellate cells in cortical layer 4 (*n* = 11 neurons), and pyramidal neurons in cortical layer 5 (*n* = 30 neurons).

**FIGURE 5:**
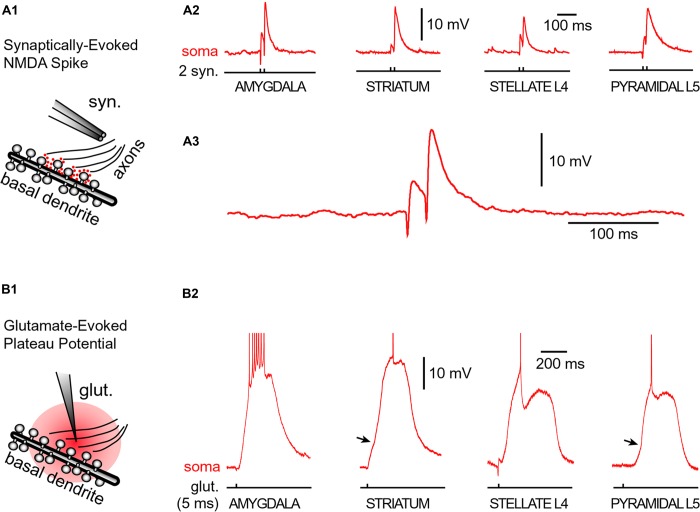
**Glutamate-mediated dendritic spikes and plateaus in spiny neurons. (A1)** Drawing depicts an experimental outline. Syn. – synaptic stimulation electrode. Red dots depict glutamate in synaptic and extrasynaptic spaces. **(A2)** Two consecutive synaptic shocks trigger classic NMDA spikes in all four types of spiny neurons. **(A3)** Amygdala NMDA spike on expanded time scale. **(B1)** Drawing depicts focal microiontophoresis of glutamate. Dendritic segment is engulfed in exogenous glutamate (red). **(B2)** Individual glutamate pulses (pulse duration = 5 ms), when delivered on spiny dendrites, produced sustained somatic depolarizations accompanied by action potential firing. APs are truncated for display. See also **Figure 3B**. Infliction points on the somatic voltage waveforms (arrows) testify to dendritic spike initiation, as determined by simultaneous dendritic voltage imaging and somatic whole-cell recordings (Milojkovic et al., 2005b).

Neurons were filled with calcium sensitive dye Oregon Green Bapta-1 (OGB-1) and synaptic stimulation electrodes were positioned in the middle portion of a thin (basilar) dendritic branch 70–90 μm away from the soma. Synaptic stimulation consisted of two shocks (pulse duration = 0.1 ms, interval = 20 ms, **Figure 5A1**, syn.). In each neuron type, we readily obtained characteristic voltage waveform of an NMDA spike (**Figures 5A2,A3**, soma). When synaptic stimulation electrodes were replaced by glutamate iontophoresis (**Figure 5B1**, pulse duration = 5 ms), each neuron type produced characteristic sustained plateau depolarizations crowned by AP firing (**Figure 5B2**, soma).

**FIGURE 6:**
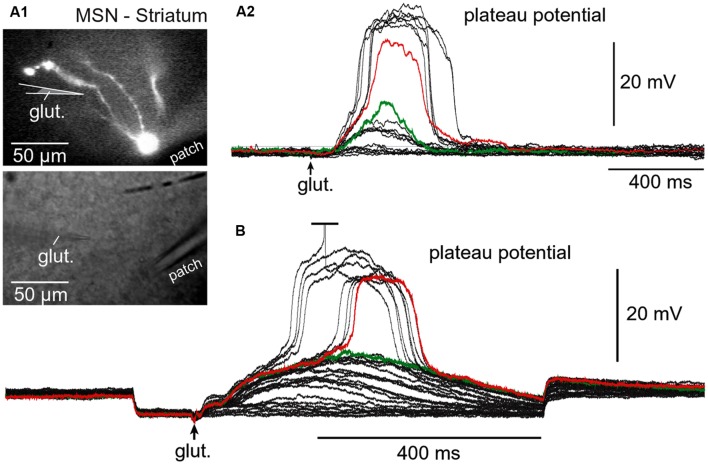
**Regenerative property of glutamate-evoked dendritic plateau potentials in neostriatum. (A)** Upper image: Neostriatal medium spiny neuron filled with OGB-1 and AF-594. Lower image: Two glass electrodes used for stimulation (glut.) and whole-cell recording (patch). **(A2)** Gradually increasing levels of the glutamate iontophoresis current produced a nonlinear membrane response (sudden jump). **(B)** Same as in **(A2)** except different cell and negative current was injected into the cell body to block action potential firing. Green indicates subthreshold and red indicates threshold responses.

Regenerative properties of glutamate-evoked dendritic plateau potentials were revealed when a series of gradually increasing glutamatergic stimuli was applied on the same dendrite (**Figure 6A1**). The transition from subthreshold to suprathreshold response (**Figure 6A2**, red trace) is attributed to the negative slope conductance in the current–voltage profile of the dendritic NMDA conductance (Schiller et al., 2000; Korogod et al., 2002; Rhodes, 2006; Major et al., 2013; Bressloff and Newby, 2014). It has been also postulated that cessation or reversal of the glutamate transport from extracellular spaces into glial processes may contribute to the abrupt transitions from subthreshold to suprathreshold response (Oikonomou et al., 2012). Regardless of the exact mechanism, the nonlinear membrane responses (abrupt transitions) were regularly observed in all four neuron subtypes during focal glutamate applications (**Figure 6B**, transition from green trace to red trace). We concluded that (1) spiny neurons of the amygdala, (2) medium spiny neurons of striatum, (3) cortical layer 4 stellate cells, and (4) cortical layer 5 pyramidal neurons process afferent glutamatergic inputs using one unified basic principle. Each neuron subtype is equipped with relatively short primary dendrites (basilar), directly attached to the soma (**Figure 2**). This morphology allows for an efficient transfer of depolarizing currents from mid dendritic segments to the soma resulting in ~20 mV somatic depolarizations (Oakley et al., 2001; Milojkovic et al., 2004, 2005a). The primary (basilar) dendrites of spiny neurons carry high density of dendritic spines, which yields to a high density of AMPA and NMDA receptors (**Figure 4**). The density of dendritic glutamatergic receptors in all spiny neurons is sufficient to support dendritic NMDA spikes (**Figure 5A**) and glutamate-mediated dendritic plateau potentials (**Figures 5B** and **6**).

### DENDRITIC UP STATES

#### Dendritic UP state in one dendrite

We do not know what causes cortical and striatal networks to turn ON and OFF on a definite temporal scale, resulting in alternating periods of high glutamatergic supply (UP state) followed by the absence of glutamatergic input (DOWN state; Wilson and Groves, 1981; Volgushev et al., 2006; Wilson, 2008). Although we do not know what causes cortical and striatal UP states (network UP states), we might be able to explain the neuronal processes which occur in dendrites of cortical and striatal neurons during such states. Here we propose that somatic voltage waveforms in spiny neurons (**Figure 3A**) are determined by dendritic UP states. The neuronal cell body shifts from a DOWN to UP state after the generation of the dendritic plateau potential (Milojkovic et al., 2004). The cell body stays in the UP state as long as the dendritic plateau lasts. The voltage waveform (sustained somatic depolarization) collapses in the cell body after the collapse of the dendritic plateau potential (Milojkovic et al., 2005a). In this way, the slow component of the somatic signal during each UP state is just a mere reflection of a flamboyant integration process occurring somewhere in the dendritic tree (Milojkovic et al., 2005a; Antic et al., 2010). This “flamboyant” integration process (dendritic plateau potential) needs to take place in only one basal branch to be a successful driver of the neuronal UP state (Milojkovic et al., 2004, 2005a, 2007).

#### Dendritic UP states occurring simultaneously in two dendrites

With thousands of synaptic contacts distributed on the basilar dendritic tree of cortical and striatal spiny neurons (Larkman, 1991; Benavides-Piccione et al., 2006; Elston et al., 2009; Garcia et al., 2010), it is likely that two or more primary (basilar) dendrites may experience glutamate-mediated plateau potentials at the same moment of time. The likelihood of coincident UP states in two and more dendrites belonging to the same neuron is high during a vigorous network activity, during elevated levels of attention or motivation, or in the face of an intense computational task. A very potent glutamatergic drive is achieved during slow wave sleep, at each “UP” phase (**Figure 3**).

**FIGURE 7:**
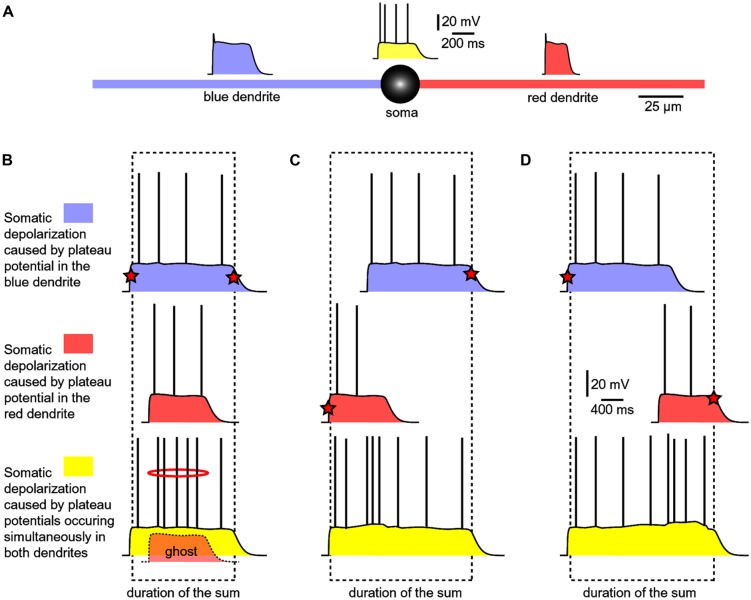
**Summation of dendritic plateau potentials on the soma. (A)** Schematic diagram of a spiny neuron representing membrane potential transients occurring simultaneously on two basal dendrites and soma. Each column/panel **(B–D)** represents one experimental sequence. Each experimental sequence has three steps. In the first step, only blue dendrite received glut. stimulus (top trace). In the second step, only red dendrite received glut. stimulus (middle trace). In the third step, both dendrites received glut. stimuli (bottom trace). Dashed rectangle marks the beginning and the end of the somatic plateau potential in the bottom trace. Red star marks the summand which contributes to the leading edge or finishing edge of the sum (yellow trace). **(B)** The red plateau starts and finishes during the plateau phase of the blue plateau. “Ghost” potential is a copy of the red trace superimposed on the bottom trace with preserved timing. The red plateau is completely “eclipsed” by the longer blue plateau, as indicated by the “ghost” potential. **(C)** The red plateau starts before the blue plateau. Duration of the sum is determined by the onset of red and the collapse of blue plateau. **(D)** The blue plateau finishes before the red plateau phase is over. Duration of the sum is thus determined by the onset of blue and the collapse of red plateau.

Experiments performed with two glutamate iontophoresis pipettes positioned on two basal dendrites were used to model dendritic spikes occurring in two basal branches at the same moment of time (Oikonomou et al., 2012). Each glutamatergic stimulus (intensity, duration) was set to trigger a dendritic plateau potential in its respective branch (**Figure 7A**). The experimental paradigm consisted of three successive steps: blue dendrite alone, red dendrite alone and both dendrites at the same time (**Figure 7B**). All traces shown in **Figures 7B–D**, represent somatic voltage waveforms. Based on the amplitude of the slow component of the somatic voltage waveform, an observer cannot readily distinguish if dendritic UP state occurred in one branch (blue or red) versus two branches simultaneously (yellow).

If a shorter dendritic plateau potential (**Figure 7B**, red) was set to occur within the plateau phase of a longer plateau potential (blue), then the shorter event would completely be “eclipsed” by the longer event upon summation (yellow). Because the amplitude of the somatic voltage waveform (slow component) was the same before (blue and red) and after summation (yellow), the only clue about the occurrence of the shorter dendritic spike (**Figure 7B**, ghost) comes from a moderate increase in AP firing (**Figure 7B**, red ring). In respect to the somatic depolarization envelope, the shorter plateau potential (red) is, in a sense, “eclipsed” by a longer dendritic plateau (blue).

In order to become a “visible” component of the sum (yellow), the red dendritic UP state must occur before the onset of the blue dendritic UP state (**Figure 7C**), or after the collapse of the blue dendritic UP state (**Figure 7D**). As long as two dendritic UP states partially overlap in time, the resulting waveform (the sum) appears as one continuous UP state in the soma (**Figures 7C,D**, yellow). One important conclusion of experiments performed with two glutamate releasing electrodes on two basal branches (**Figure 7**) is that during a neuronal UP state, the slow component of the somatic voltage waveform does not reveal the number of basal dendrites experiencing glutamate-mediated plateau potentials (Oikonomou et al., 2012). Only when these potentials are separated (shifted) in time, so that their profiles (plateau phases) no longer overlap, the cell body can “experience” two dendritic plateau potentials arriving from two basal dendrites as two separate events (Oikonomou et al., 2012, their Figure 9).

### DETECTORS OF STRONG NETWORK ACTIVITY

A successful synchronization of the firing activity among neurons would require that members of a neuronal ensemble enter UP state at the same moment of time (**Figure 1B**). To become eligible for inclusion into a functional neuronal ensemble, a spiny neuron must quickly, and reliably switch from a DOWN to an UP state, remain in the depolarized UP state as long as necessary, and quickly abandon the UP state when a percept is formed or expired. Several lines of evidence listed below (Sections Efficient Depolarization of the Cell Body, Dependence on the Surplus Glutamate, Duration of Sustained Depolarization, and Dendritic Spines and Glial Processes) suggest that glutamate-mediated dendritic plateau potentials may serve as detectors of significant or meaningful network activity, and may underlie the neuronal voltage waveforms recorded *in vivo* (**Figure 3A**).

#### Efficient depolarization of the cell body

**FIGURE 8:**
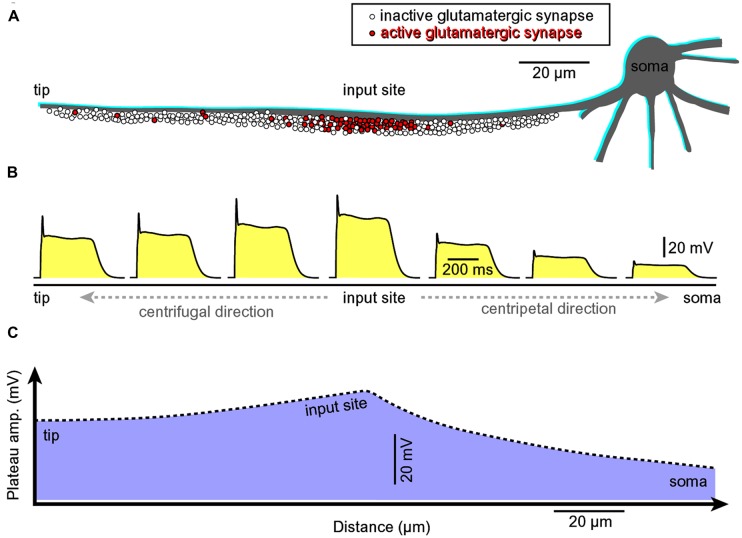
**Asymmetric propagation of dendritic plateau potential. (A)** Schematic drawing of a spiny basal dendrite. Red dots denote glutamatergic afferents actively releasing glutamate at this moment of time. **(B)** Voltage waveform of the glutamate-induced plateau potential simultaneously viewed at seven different sites along the spiny dendrite (Milojkovic et al., 2004, 2005a, 2007). **(C)** The amplitude of the slow component (plateau phase) attenuates as dendritic potential spreads passively toward the cell body (centripetal direction, Milojkovic et al., 2004; their Figures 3 and 4). However, the duration remains the same across the entire dendritic branch. The grade of attenuation is less in centrifugal direction (from the initiation site toward the dendritic tip) than in the centripetal direction. Regardless of direction (centripetal or centrifugal), the propagating plateau potentials successively open voltage-gated Ca^2+^ channels in dendritic segments, which explains why the entire dendritic branch experiences significant calcium influx during a glutamate-evoked dendritic plateau potential (even though synaptic glutamate receptor channels are activated at the input site only). This interplay between dendritic membrane potential and dendritic calcium influx in space and time was revealed by combining voltage-sensitive and calcium-sensitive multi-site recordings along the same dendritic branch (Milojkovic et al., 2007).

A glutamate-mediated dendritic plateau potential (dendritic UP state) produces enough depolarizing current to drive the cell body into a sustained depolarized state (neuronal UP state; Milojkovic et al., 2004; Augustinaite et al., 2014). The amplitude of the slow component (plateau phase) at the dendritic initiation site (**Figure 8**, input site) located in the middle of a basal dendrite (100–150 μm away from the cell body) is ~2/3 of the backpropagating AP at the same location (Milojkovic et al., 2004). The amplitude of the backpropagating AP at 100–150 μm away from the cell body is ~60 mV (Antic, 2003; Acker and Antic, 2009). Therefore, the amplitude of the dendritic plateau potential is ~40 mV. The amplitude of the dendritic plateau potential decreases gradually as dendritic voltage transient spreads passively into the cell body (**Figure 8A**, centripetal direction of propagation), resulting in a ~20 mV somatic depolarization (Milojkovic et al., 2004, 2005a,b, 2007; Major et al., 2008). The amplitude of the sustained somatic depolarization (neuronal UP state) depends on the physical location of the input site. It is lessened if its glutamatergic input is moved more distally, away from the cell body (Milojkovic et al., 2004, their Figure 1; Major et al., 2008; Augustinaite et al., 2014; Jadi et al., 2014).

#### Dependence on the surplus glutamate

Cortical or striatal UP and DOWN states are caused by the alternating presence and absence of activity in excitatory neuronal network (Wilson and Kawaguchi, 1996; Sanchez-Vives and McCormick, 2000; Fellin et al., 2004; Poskanzer and Yuste, 2011). During periods of greater network activity, a significant glutamatergic input impinges on individual neurons, causing these neurons to enter the UP state. Transitions to the UP state are robust phenomena that accurately reflect the underlying structure of consistent increases in afferent input over a limited time period. There are no transitions back to the DOWN state until the excitatory glutamatergic input is reduced (Wolf et al., 2005). Similarly to UP states, dendritic plateau potentials occur only if dendritic shafts and associated extrasynaptic NMDA receptors are surrounded by a surplus of glutamate ions/molecules (Suzuki et al., 2008; Chalifoux and Carter, 2011; Oikonomou et al., 2012). In summary, a surplus of glutamate (spillover) occurs during network UP states (Lambe and Aghajanian, 2006; Poskanzer and Yuste, 2011). A surplus of glutamate in the extrasynaptic space triggers dendritic plateau potential (Suzuki et al., 2008; Chalifoux and Carter, 2011; Oikonomou et al., 2012).

#### Duration of sustained depolarization

There is a strong similarity between UP states and dendritic plateau potentials regarding the duration of sustained depolarization. Both network UP states and glutamate-mediated dendritic plateau potentials last several hundred milliseconds (Figures 3 and 5). We think that a continued presence of glutamate molecules bound to NMDA receptors in synaptic and more importantly in extrasynaptic spaces is critical for the maintenance of the UP state. Unlike a classic sodium spike which is terminated by the inactivation of Na^+^ and strong activation of K^+^ currents, the glutamate-mediated dendritic plateau potential is terminated by unbinding of glutamate and weak activation of K^+^ currents (Cai et al., 2004). Glutamate unbinding from dendritic NMDA receptor channels is a slower process because it takes place inside the glutamate pond created by repetitive synaptic stimulation (Oikonomou et al., 2012), hence explaining the prolonged plateau phases of these dendritic events (Milojkovic et al., 2004, 2005a, 2007).

#### Dendritic spines and glial processes

CNS spiny neurons possess an inherent mechanism for generation of dendritic UP states, which is based on anatomical and functional relations between dendritic spines and glial processes interposed between dendritic spines. The growth of dendritic spines endows spiny neurons with four cardinal features:

***High density of impinging glutamate-releasing axon terminals (Figure 4).*** Note that an ample supply of glutamate is essential for dendritic plateau potentials. The amount of glutamate required to drive a plateau potential can only be obtained by repetitive synaptic stimulation or glutamate iontophoresis (Milojkovic et al., 2004; Major et al., 2008; Suzuki et al., 2008; Augustinaite et al., 2014). However, sequential glutamate uncaging on 10 dendritic spines cannot supply enough glutamate to create a glutamate pond (Losonczy et al., 2008; Remy et al., 2009; Branco and Hausser, 2011), and this may be the reason why these experiments did not yield glutamate-mediated dendritic plateau potentials lasting hundreds of milliseconds.

***High density of NMDA receptor-channels.*** High density of dendritic NMDA conductance is essential for the generation of glutamate-mediated dendritic regenerative potentials (Schiller et al., 2000; Rhodes, 2006; Major et al., 2013). Without any doubt, dendritic spines increase the surface area for the insertion of synaptic and extrasynaptic NMDA receptors resulting in a significantly greater NMDA conductance per dendritic branch (**Figure 4**), thus providing the critical requirement for dendritic NMDA spike initiation (Schiller et al., 2000; Rhodes, 2006; Major et al., 2013).

***High density of glial processes surrounding dendritic branch.*** Growth of spines creates space for glial processes to grow in between dendritic spines (**Figure 4A**, glia). Strategic positioning of glial processes between the populations of synaptic and extrasynaptic NMDA receptors (**Figure 4B**), provides astrocytes with a mechanism to gate neuronal transitions from DOWN to UP state (Lambe and Aghajanian, 2006; Poskanzer and Yuste, 2011).

***Spine necks provide for the separation between two principal modes of synaptic transmission.*** (a) Subthreshold (dendritic EPSP) and (b) suprathreshold dendritic response (plateau potential). Dendritic spines create a physical separation between synaptic and extrasynaptic NMDA receptor-channels (**Figure 4A**). The length of the spine neck represents a physical distance that spillover glutamate has to travel in order to reach extrasynaptic NMDA receptors located on the dendritic shaft (**Figure 4B**). If glutamate molecules “survive” the trip from the releasing axons to the surface of the dendritic shaft (**Figure 4A**), then a subthreshold dendritic potential (**Figure 4B**, EPSP) is converted into a suprathreshold membrane response termed “dendritic plateau potential” (**Figure 4B**).

The presence of glutamate molecules is not simply a permissive factor for the initiation of a glutamate-mediated dendritic plateau potential (dendritic UP state). Instead, a nonlinear buildup of glutamate is probably the primary mechanism of the observed voltage jump from subthreshold voltage transient to a full-blown spike (**Figure 6B**), see also (Schiller et al., 2000; Milojkovic et al., 2004, 2005a; Oikonomou et al., 2012). It can be said that dendritic plateau potentials have a “glutamate threshold” (Milojkovic et al., 2005b; Major et al., 2008; Polsky et al., 2009).

**FIGURE 9:**
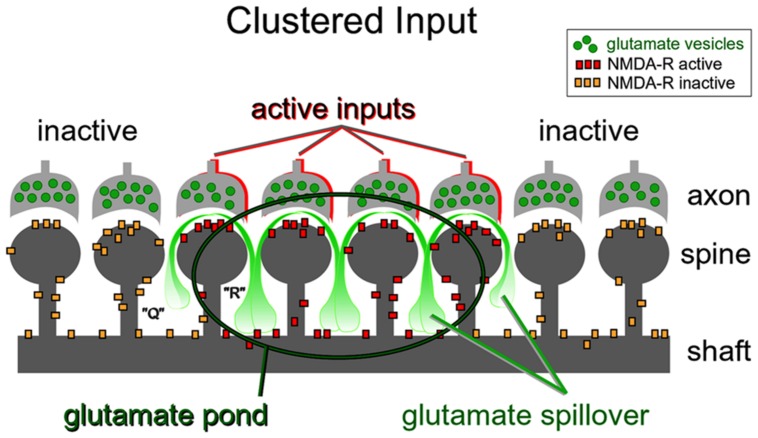
**Dendritic plateau potentials improve the efficacy of the conversion of excitatory inputs into sustained somatic depolarizations.** Active glutamatergic inputs are spatiotemporally “clustered” onto a dendritic segment. Glutamate spillover is more efficient between two neighboring active inputs (space “R”) than between an active and inactive input (space “Q”). Glutamate spillover may overwhelm glial processes located between neighboring active inputs causing a local accumulation of glutamate in the extracellular space (glutamate pond). Inside the glutamate pond, both synaptic and extrasynaptic NMDA receptors are fully activated, thus creating favorable conditions for firing of a glutamate-mediated dendritic plateau potential.

***Nonlinear build-up of glutamate in the extracellular space (“glutamate threshold”).*** During intense network activity, many converging glutamatergic preterminals (**Figure 4**, axon) are activated repetitively by bursts of action potentials traveling through axonal lines of communication (Lisman, 1997). Repetitive synaptic input is a key requirement for the dendritic UP state (Milojkovic et al., 2004; Oikonomou et al., 2012). At some point during repetitive synaptic stimulation, an ensuing glutamatergic drive overwhelms the ability of glial processes to absorb the spilled glutamate. The “glutamate threshold” is reached when glia is no longer able to cope with repetitive glutamatergic inputs arriving in a confined space at the same moment of time (**Figure 9**). For a brief period of time the dendritic segment is surrounded by a surplus of glutamate (**Figure 9**, “glutamate pond”). During such an overwhelming glutamatergic stimulus, the dendritic spike cannot be perturbed by negative voltage pulses (Oikonomou et al., 2012; their Figure 5).

***Repetitive inputs.*** In the process of dendritic spike initiation, the primary role of the repetitive glutamatergic input is not a local depolarization needed to cross the voltage threshold (Polsky et al., 2009), but instead multiple shocks are necessary to reverse glial function from glutamate uptake to glutamate release (Parpura et al., 1994). In glutamate uncaging experiments, the NMDA spike is initiated only when experimenters select neighboring dendritic spines (Losonczy et al., 2008; Remy et al., 2009; Branco and Hausser, 2011). The reason for this is contained in the spatial arrangement of the participating ultrastructures. Glial processes interposed between two active dendritic spines (**Figure 9**, space “R”) are more likely to reverse glial function from glutamate uptake to glutamate release (Parpura et al., 1994), than the glial processes interposed in between active and inactive spines (**Figure 9**, space “Q”). In summary, the new evidence (Major et al., 2008; Polsky et al., 2009; Oikonomou et al., 2012) supports the notion that dendritic spikes in glutamate uncaging experiments do not arise from summation of voltage alone, but rather from summation of three glutamate sources: (1) uncaged glutamate; (2) synaptically released glutamate triggered by the presence of uncaged glutamate; and (3) glutamate released from glia stimulated by the uncaged glutamate (Min and Nevian, 2012).

## CONCLUDING REMARKS

Although a diffuse glutamatergic input distributed across an entire dendritic tree may be used by neurons for the detection of strong network activity and conversion of such activity into a sustained plateau depolarization (UP state; Shu et al., 2003), a more effective mechanism is the mechanism based on the convergence of synaptic inputs onto one dendritic branch (Mel, 1993) and induction of a long-lasting glutamate-mediated regenerative dendritic potential (Milojkovic et al., 2004). Excitatory glutamatergic inputs confined to a single dendrite can profoundly influence the neuronal output of layer 5 pyramidal neurons in brain slices (**Figure 3**). “A common preconception about central nervous system neurons is that thousands of small postsynaptic potentials sum across the entire dendritic tree to generate substantial firing rates” (Milojkovic et al., 2004). Contrary to this common presumption, a brief glutamatergic stimulation delivered in a restricted part of the basilar dendritic tree invariably produces sustained plateau depolarizations of the cell body, accompanied by bursts of action potentials (Milojkovic et al., 2004, 2005a). Glutamatergic inputs converging on a narrow segment of a single dendritic branch is sufficient input for generation of a somatic depolarization, which strongly resembles neuronal UP state (Milojkovic et al., 2004, 2005a; Antic et al., 2007, 2010). Plotkin et al. (2011), arrived at an identical conclusion studying striatal medium spiny neurons.

All spiny neurons of the mammalian telencephalon, including pyramidal layers 2–6 and stellate layer 4 neurons of the cerebral cortex, medium spiny neurons of the neostriatum, amygdala, and nucleus accumbens are well positioned to detect multiple patterns of highly selected inputs, perhaps as few as 50–100 inputs from each afferent structure. Spiny neurons integrate inputs over a relatively large time window and are probably detecting the co-occurrence of signature patterns of afferent inputs relating context, emotion, and working memory (Bar-Gad et al., 2003; Wolf et al., 2005). Glutamate-mediated dendritic plateau potentials are ideally built to provide neurons with a relatively large integration window lasting several hundred milliseconds (Milojkovic et al., 2004, 2005a). This temporal window is a critical determinant of the “ensemble period” as depicted in **Figure 1**. The size (number of cell-members) and power (ability to drive the organism toward a distinct behavior) of a neuronal ensemble both depend on the ability of each cell-member to remain in a sustained depolarized state. The amount of time each neuron spends in a sustained depolarized UP state is the product of the duration of a dendritic plateau potential and the number of dendritic branches experiencing plateau potentials overlapping in time (**Figure 7**, *duration of the sum*).

Initially, *in vivo* recordings were unable to provide evidence that dendritic NMDA spikes or plateau potentials occur in living animals (Waters and Helmchen, 2006; Waters, 2007; Varga et al., 2011). However, recent advancements in technology have produced experimental evidence in favor of dendritic spikes in anesthetized and behaving animals (Lavzin et al., 2012; Smith et al., 2013). What’s more, a recent study has found NMDA-dependent dendritic calcium signals locked to neuronal UP states (Hill et al., 2013). All in all, experimental studies reporting the occurrence on dendritic NMDA spikes *in vivo* may accumulate with time (Grienberger et al., 2014; Palmer et al., 2014).

Whether spiny neurons have evolved a dendritic mechanism for detecting activity of neuronal ensembles (**Figure 1**) and joining the active ensemble (transition to UP state) remains to be further investigated *in vivo*. Nevertheless, there is little doubt that in all telencephalic neurons with dendritic spines (spiny neurons), dendritic NMDA spikes and glutamate-mediated dendritic plateau potentials represent the dominant forms of dendritic integration.

## Conflict of Interest Statement

The authors declare that the research was conducted in the absence of any commercial or financial relationships that could be construed as a potential conflict of interest.
